# Association of the Interaction Between Mammographic Breast Density, Body Mass Index, and Menopausal Status With Breast Cancer Risk Among Korean Women

**DOI:** 10.1001/jamanetworkopen.2021.39161

**Published:** 2021-12-23

**Authors:** Thi Xuan Mai Tran, Seong-Geun Moon, Soyeoun Kim, Boyoung Park

**Affiliations:** 1 Department of Preventive Medicine, Hanyang University College of Medicine, Seoul, Republic of Korea; 2 Department of Health Sciences, Hanyang University College of Medicine, Seoul, Republic of Korea

## Abstract

**Importance:**

Evidence suggests that breast density and body mass index (BMI) are strong breast cancer risk factors; however, their interactive associations are unknown. Elucidation of these interactive associations may help to increase understanding of the causes of breast cancer and find effective interventions for susceptible subgroups.

**Objective:**

To explore the association of the interaction of mammographic breast density and BMI with breast cancer risks among premenopausal and postmenopausal women.

**Design, Setting, and Participants:**

This prospective observational cohort study used population-based data of the Korean National Cancer Screening Program embedded in the National Health Insurance Service database to evaluate the breast cancer risk of 3 248 941 premenopausal cancer-free women and 4 373 473 postmenopausal cancer-free women aged 40 years or older who underwent mammographic screening between January 1, 2009, and December 31, 2013, and were followed up until December 31, 2018. Statistical analysis was performed from June 1 to July 15, 2021.

**Exposures:**

Breast Imaging Reporting and Data System (BI-RADS)–defined breast density (with a scale from 1 to 4, where 1 indicates almost entirely fat, 2 indicates scattered fibroglandular densities, 3 indicates heterogeneously dense tissue, and 4 indicates extremely dense tissue) and BMI levels classified according to the World Health Organization Asia-Pacific Region classification.

**Main Outcomes and Measures:**

Adjusted relative risk (aRR) of breast cancer during the follow-up period and interactions in additive and multiplicative scales. The study end point was the development of breast cancer.

**Results:**

Of 3 248 941 premenopausal women (mean [SD] age, 44.6 [4.3] years) and 4 373 473 postmenopausal women (mean [SD] age, 59.6 [8.4] years) aged 40 years or older, 34 466 cases of breast cancer were identified among the premenopausal women, and 30 816 cases of breast cancer were identified among the postmenopausal women. Increased breast density was associated with an increased risk of breast cancer in both premenopausal and postmenopausal women across the BMI categories. Among premenopausal women, those in BI-RADS category 4 had an approximately 2-fold higher risk of breast cancer irrespective of BMI (all women: aRR, 2.36 [95% CI, 2.24-2.49]; underweight: aRR, 1.80 [95% CI, 1.25-2.59]; normal weight: aRR, 2.10 [95% CI, 1.93-2.28]; overweight: aRR, 2.47 [95% CI, 2.27-2.68]; obese: aRR, 2.87 [95% CI, 2.49-3.32]) than those with underweight status and in BI-RADS category 1. However, an association between BMI and the risk of breast cancer was found only in the postmenopausal women in all breast density categories compared with underweight women with BI-RADS category 1 (BI-RADS category 4, all women: aRR, 2.91 [95% CI, 2.78-3.04]; underweight: aRR, 2.74 [95% CI, 1.89-3.98]; normal weight: aRR, 3.05 [95% CI, 2.82-3.30]; overweight: aRR, 2.85 [95% CI, 2.67-3.04]; obese: aRR, 2.52 [95% CI, 2.22-2.88]). When the combined associations of breast density and BMI with the risk of breast cancer were considered, a high breast density and high BMI had a significant positive interaction on the additive scale for both premenopausal and postmenopausal women, especially the latter (premenopausal women: adjusted relative excess risk due to interaction, 0.53 [95% CI, 0.35-0.71]; postmenopausal women: adjusted relative excess risk due to interaction, 1.68 [95% CI, 1.26-2.10]).

**Conclusions and Relevance:**

This study suggests that breast density and BMI interact synergistically to augment breast cancer risk, with a stronger association found among postmenopausal women. Both factors should be incorporated into risk stratification in a population-based screening for public health significance. Women with overweight or obesity and dense breast tissue might benefit from tailored early screening strategies to detect breast cancer.

## Key Points

**Question:**

Is the interaction between mammographic breast density and overweight or obesity associated with breast cancer risk, and if so, does any association vary according to menopausal status?

**Findings:**

In a cohort study of 3 248 941 premenopausal and 4 373 473 postmenopausal women aged 40 years or older screened for breast cancer, a positive additive interaction between high breast density and high body mass index was associated with an increased risk of breast cancer, especially among women in the postmenopausal period.

**Meaning:**

This study suggests that women with overweight or obesity and dense breast tissue might benefit from tailored breast cancer screening strategies; these 2 factors should be incorporated into risk stratification in population-based breast cancer screening.

## Teaser

This cohort study uses data from the Korean National Cancer Screening Program to evaluate the association of the interaction of mammographic breast density and body mass index with breast cancer risks among premenopausal and postmenopausal women.

## Introduction

Female breast cancer is the most commonly diagnosed cancer, accounting for 11.7% of all new cases of cancer in 2020.^1^ Of the various risk factors for breast cancer, dense breast tissue has become an important issue, with a strong association between dense breast tissue and increased breast cancer risk regardless of menopausal status.^2,3^ Meanwhile, body mass index (BMI; calculated as weight in kilograms divided by height in meters squared) appears to have the opposite association with breast cancer risk according to menopausal status. A high BMI had no association or was associated with a reduced breast cancer risk,^4^ whereas increased adiposity was positively associated with increased postmenopausal breast cancer.^5^

Although dense breast tissue and obesity were individually proven to be strong risk factors for breast cancer, controversy exists regarding the magnitude of the combined associations of these 2 factors with breast cancer risk, in addition to heterogeneity of the associations according to menopausal status.^6,7,8,9^ In particular, whether the combined association of breast density and BMI with breast cancer risk varies according to menopausal status has yet to be comprehensively examined, to our knowledge. Some findings suggest that women with overweight or obesity and dense breast tissue may have a high risk of breast cancer regardless of menopausal status.^10,11,12^ However, 1 pooled analysis from 4 case-control studies reported minimal modification by BMI of the associations of breast density with breast cancer risk.^13^

Mammographic breast density and BMI could be measured during health examinations, and evaluating their joint associations could provide information on future breast cancer risk during screening and consequently help identify women at an elevated risk of breast cancer. Thus, this study aimed to explore the interaction of breast density and BMI and risk of breast cancer according to menopausal status.

## Methods

### Study Design and Population

In this cohort study, we used data from the Korean National Cancer Screening Program embedded in the National Health Insurance Service (NHIS) database.^14^ In Korea, the NHIS provides free biennial mammography breast cancer screening for women aged 40 years or older. Ascertainment of incident breast cancer cases was obtained by linking the database to the medical use database of the NHIS. Breast cancer cases were defined by the *International Statistical Classification of Diseases and Related Health Problems, Tenth Revision* codes for invasive breast cancer (C50.0-C50.9) and ductal carcinoma in situ (D05.0-D05.9), in combination with the catastrophic illness code.^15^ Screening results and questionnaire data were transferred to the NHIS database after obtaining written informed consent from the women. Our study was approved by the institutional review board of the Hanyang University College of Medicine. In addition, permission to use the NHIS database was approved by the National Health Insurance Sharing Service system, and deidentified data were available to the researchers. This study followed the Strengthening the Reporting of Observational Studies in Epidemiology (STROBE) reporting guideline.

**Figure 1.**
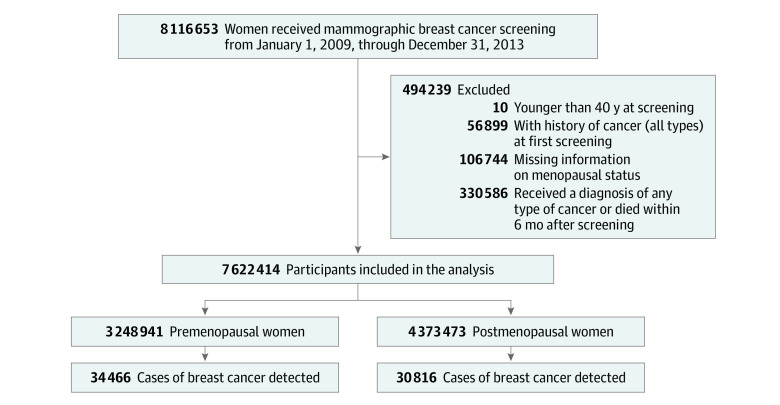
Flow Diagram of the Selection of the Eligible Population

We used data of women who underwent mammography screening between January 1, 2009, and December 31, 2013. Information on breast cancer incidence was followed up until December 31, 2018. When a woman underwent mammographic screening more than once, information on the first screening was used. Among 8 116 653 women who underwent screening for breast cancer between 2009 and 2013, those who underwent screening at younger than 40 years of age, those with any cancer history before the date of mammographic screening, or those with missing information on menopausal status, BMI, or breast density were excluded. In addition, participants who received a diagnosis of any type of cancer or died within 180 days after breast cancer screening were excluded to prevent possible inclusion of prevalent cancer cases; the remaining 7 622 414 women were composed of 3 248 941 premenopausal and 4 373 473 postmenopausal women at baseline (Figure 1).

### Measurement of Mammographic Breast Density

Mammographic breast density was evaluated using the Breast Imaging Reporting and Data System (BI-RADS) classification of the American College of Radiology. The BI-RADS breast density was read by the radiologist at each screening center. The BI-RADS classification has been used to categorize breast density in the Korean National Cancer Screening Program since 2009. The BI-RADS mammographic breast density comprises 4 categories: 1 indicates almost entirely fat, 2 indicates scattered fibroglandular densities, 3 indicates heterogeneously dense tissue, and 4 indicates extremely dense tissue.

### Measurement of BMI

During health examinations, height and weight were measured by trained nurses and used to calculate BMI. This study used the BMI cutoff points according to the World Health Organization Asia-Pacific classification.^16^ Body mass index was categorized into the following 4 groups: underweight (<18.5), normal weight (18.5 to <23.0), overweight (23.0 to <27.5), and obese (≥27.5).

### Measurement of Other Covariates

Information on other covariates was collected using standardized questionnaires during health examination and cancer screening. The questionnaires were distributed by nurses and self-reported by participants. The questionnaires measured information regarding health behaviors, including drinking, smoking, physical activity, family history of cancer and other major chronic diseases, and the reproductive physiological processes of all of the participants. In our analysis, we considered the following factors as covariates: participants’ age at screening, history of smoking, number of days of alcohol consumption per week, family history of breast cancer among first-degree relatives, history of benign breast disease, age at menarche, number of children, duration of breastfeeding, history of breastfeeding, use of oral contraceptives, and history of hormone replacement therapy.

### Statistical Analysis

Statistical analysis was performed from June 1 to July 15, 2021. The study end point was the development of breast cancer. The association among BMI levels, BI-RADS breast density categories, and breast cancer risk was presented in terms of relative risks (RRs) using Poisson regression models, considering the rare events of breast cancer. The associations between BMI and breast cancer risk according to BI-RADS density classification and between BI-RADS density classification and breast cancer risk according to BMI were also evaluated. In addition, the combined associations of BMI and the BI-RADS density classification with breast cancer risk were presented using a Poisson regression model. All analyses were stratified by menopausal status and adjusted for the covariates as mentioned.

The interactions between breast density classification and BMI on the additive and multiplicative scales were examined in association with breast cancer risk. The additive interactions were assessed using relative excess risk due to interaction (RERI), which was computed as RERI_RR_ = RR_11_ − RR_10_ − RR_01_ + 1. An RERI value of 0 indicated no additive interaction, a positive value indicated a super-additive or positive interaction, and a negative value indicated a subadditive (negative) interaction. The multiplicative interactions were assessed using the ratio of RRs: RR_11_/(RR_10_ × RR_01_). A multiplicative interaction value of more than 1 indicated a positive interaction, while a multiplicative interaction value of less than 1 indicated a negative interaction.^17^ Additive interaction is present when the combined association of BMI and breast density is larger than the sum of the individual associations. Multiplicative interaction was estimated to evaluate whether the combined association of BMI and breast density with breast cancer risk was larger than the product estimated association of BMI alone and breast density alone.^17^

A 2-sided *P* < .05 was considered statistically significant. The analyses were performed using SAS, version 9.4 (SAS Institute Inc).

## Results

### Baseline Characteristics of the Study Participants

In this cohort of 3 248 941 premenopausal cancer-free women (mean [SD] age, 44.6 [4.3] years) and 4 373 473 postmenopausal cancer-free women (mean [SD] age, 59.6 [8.4] years), 34 466 premenopausal women and 30 816 postmenopausal women subsequently received a diagnosis of breast cancer (Table 1; eTable 1 in the Supplement). The proportion of premenopausal women with overweight or obesity was 46% among those who did not develop breast cancer (1 490 486 of 3 214 475) and 45% among those who developed breast cancer (15 578 of 34 466). Among postmenopausal women, 63% of those without breast cancer (2 740 424 of 4 342 657) and 66% of those with breast cancer (20 304 of 30 816) had overweight or obesity. Among both premenopausal and postmenopausal women, the proportion of those in BI-RADS categories 3 and 4 was higher among those who developed breast cancer than among those who did not (82% [28 394 of 34 466] vs 73% [2 355 508 of 3 214 475] among the premenopausal women and 46% [14 015 of 30 816] vs 30% [1 300 682 of 4 342 657] among the postmenopausal women; *P* < .001).

**Table 1.** Characteristics of the Study Participants at Screening

Characteristic	Premenopausal women, No. (%)			Postmenopausal women, No. (%)		
	No (n = 3 214 475)	Yes (n = 34 466)	*P* value	No (n = 4 342 657)	Yes (n = 30 816)	*P* value
Age, mean (SD), y	44.4 (4.3)	44.8 (4.2)	<.001	60.5 (8.9)	58.6 (7.9)	<.001
BMI						
Underweight	113 533 (4)	1269 (4)	<.001	89 768 (2)	455 (2)	<.001
Normal	1 610 456 (50)	17 619 (51)		1 512 465 (35)	10 057 (33)	
Overweight	1 183 185 (37)	12 541 (36)		2 113 806 (49)	15 165 (49)	
Obese	307 301 (10)	3037 (9)		626 618 (14)	5139 (17)	
BI-RADS category for breast density						
1	256 242 (8)	1545 (5)	<.001	1 656 322 (38)	6667 (22)	<.001
2	602 725 (19)	4527 (13)		1 385 653 (32)	10 134 (33)	
3	1 380 110 (43)	15 398 (45)		993 853 (23)	10 366 (34)	
4	975 398 (30)	12 996 (38)		306 829 (7)	3649 (12)	
Family history of breast cancer among first-degree relatives						
No	3 081 810 (96)	32 290 (94)	<.001	4 148 427 (96)	28 918 (94)	<.001
Yes	61 844 (2)	1398 (4)		70 402 (2)	1068 (4)	
Unknown	70 821 (2)	778 (2)		123 828 (3)	830 (3)	
History of benign breast cancer disease						
No	2 692 649 (84)	27 021 (78)	<.001	3 714 693 (86)	24 469 (79)	<.001
Yes	289 002 (9)	4857 (14)		276 483 (6)	3709 (12)	
Unknown	232 824 (7)	2588 (8)		351 481 (8)	2638 (9)	
Age at menarche, y						
<16	2 214 574 (69)	24 649 (72)	<.001	1 510 945 (35)	13 039 (42)	<.001
≥16	957 707 (30)	9346 (27)		2 763 765 (64)	17 295 (56)	
Missing	42 194 (1)	471 (1)		67 947 (2)	482 (2)	
Parity						
Nulliparous	206 539 (6)	2972 (9)	<.001	141 770 (3)	1640 (5)	<.001
Parous	3 007 936 (94)	31 494 (91)		4 200 887 (97)	29 176 (95)	
Breastfeeding						
Never	601 274 (19)	7675 (22)	<.001	363 566 (8)	3942 (13)	<.001
Ever	2 527 652 (79)	25 626 (74)		3 910 245 (90)	26 173 (85)	
Unknown or NA	85 549 (3)	1165 (3)		68 846 (2)	701 (2)	
Oral contraceptive use						
Never	2 657 637 (83)	28 538 (83)	.42	3 468 373 (80)	24 197 (79)	<.001
Ever	421 509 (13)	4448 (13)		638 476 (15)	4777 (16)	
Unknown	135 329 (4)	1480 (4)		235 808 (5)	1842 (6)	
Smoking status						
Never	2 997 416 (93)	32 054 (93)	.02	4 134 655 (95)	29 004 (94)	<.001
Ever	209 462 (7)	2346 (7)		195 442 (5)	1708 (6)	
Unknown	7597 (0.2)	66 (0.2)		12 560 (0.3)	104 (0.3)	
Alcohol consumption						
No drinking	2 206 057 (69)	23 782 (69)	.27	3 698 715 (85)	25 757 (84)	<.001
Drinking	998 998 (31)	10 591 (31)		624 388 (14)	4914 (16)	
Unknown	9420 (0.3)	93 (0.3)		19 554 (1)	145 (1)	
Hormone replacement therapy among menopausal women						
Never	NA	NA	NA	3 119 181 (72)	20 033 (65)	<.001
Ever	NA	NA		570 366 (13)	5009 (16)	
Unknown	NA	NA		653 110 (15)	5774 (19)	

Abbreviations: BI-RADS, Breast Imaging Reporting and Data System; BMI, body mass index (calculated as weight in kilograms divided by height in meters squared); NA, not applicable.

### Associations of Breast Density With Breast Cancer Risk According to BMI

**Figure 2.**
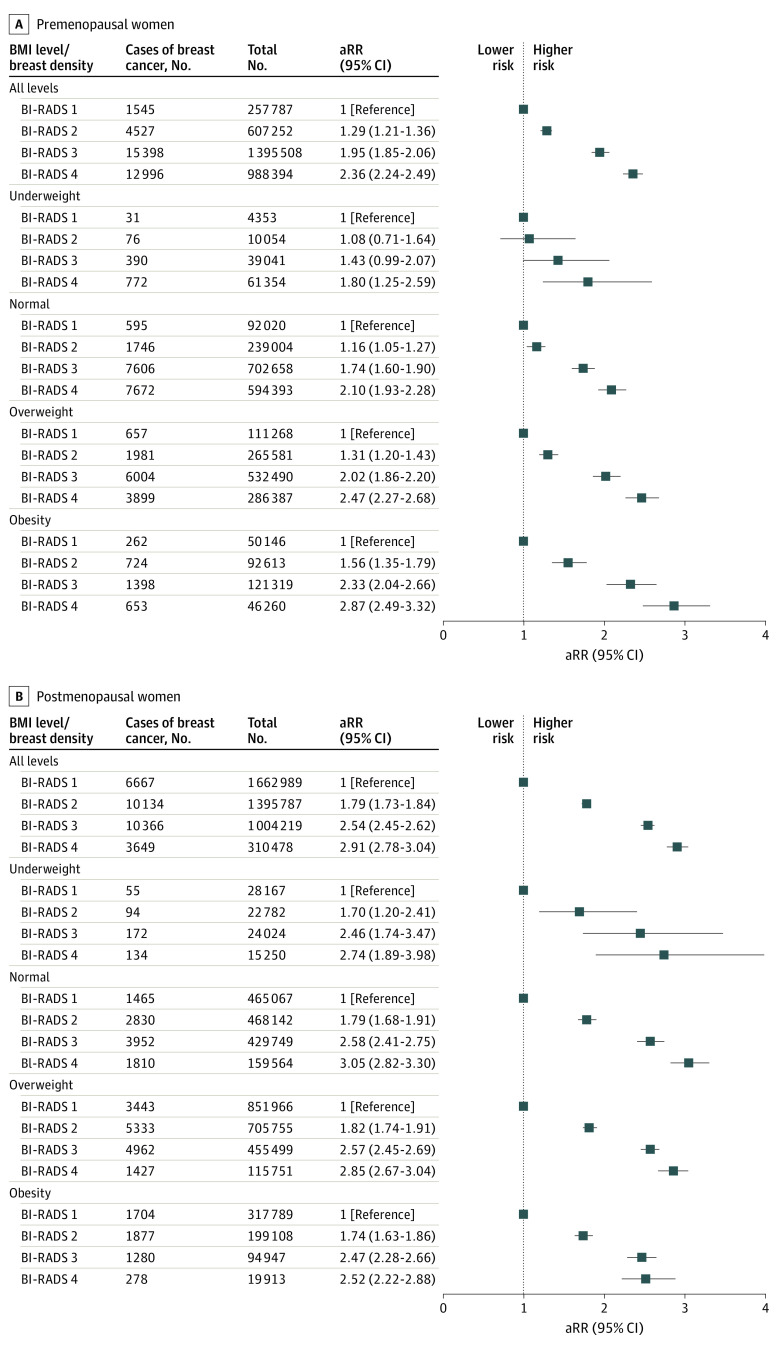
Association Between Breast Density and Breast Cancer Risk According to Body Mass Index (BMI) A relative risk greater than 1 represented a higher risk of breast cancer. Error bars indicate 95% CIs for the relative risk. aRR indicates adjusted relative risk; BI-RADS, Breast Imaging Reporting and Data System.

Increased breast density was associated with an increased risk of breast cancer for both premenopausal and postmenopausal women in total and across 4 BMI categories (Figure 2; eTable 2 in the Supplement). Among premenopausal women, those in BI-RADS category 4 had an approximately 2-fold higher risk of breast cancer irrespective of BMI (all women: adjusted RR [aRR], 2.36 [95% CI, 2.24-2.49]; underweight: aRR, 1.80 [95% CI, 1.25-2.59]; normal weight: aRR, 2.10 [95% CI, 1.93-2.28]; overweight: aRR, 2.47 [95% CI, 2.27-2.68]; obese: aRR, 2.87 [95% CI, 2.49-3.32]) than those with underweight status and in BI-RADS category 1. In addition, those with a high BMI showed associations between high BI-RADS categories and breast cancer risks. Similar associations were found among postmenopausal women, with a significant association between high breast density and breast cancer risk across the BMI categories compared with underweight women with BI-RADS category 1 (BI-RADS category 4, all women: aRR, 2.91 [95% CI, 2.78-3.04]; underweight: aRR, 2.74 [95% CI, 1.89-3.98]; normal weight: aRR, 3.05 [95% CI, 2.82-3.30]; overweight: aRR, 2.85 [95% CI, 2.67-3.04]; obese: aRR, 2.52 [95% CI, 2.22-2.88]).

### Associations of BMI With Breast Cancer Risk According to Breast Density

**Figure 3.**
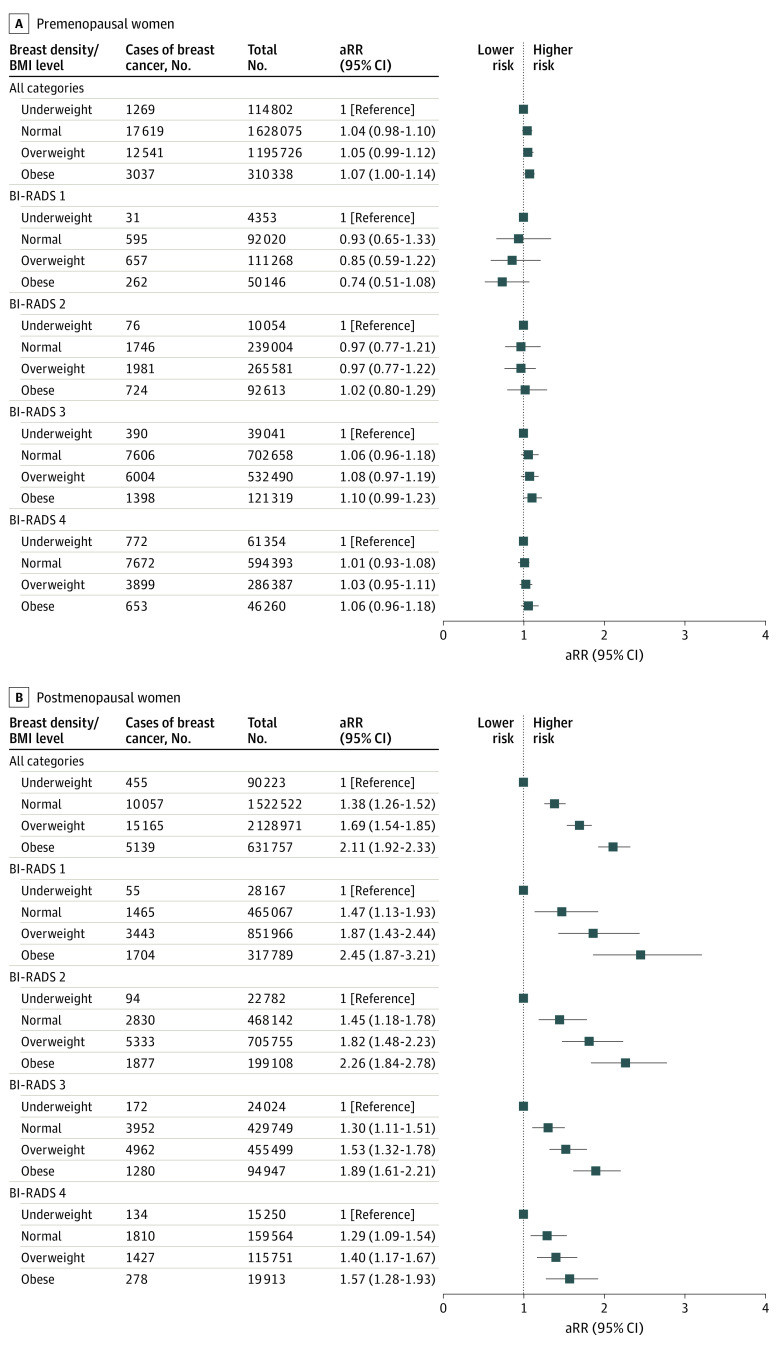
Association Between Body Mass Index (BMI) and Breast Cancer Risk According to Breast Density A relative risk greater than 1 represented a higher risk of breast cancer. Error bars indicate 95% CIs for the relative risk. aRR indicates adjusted relative risk; BI-RADS, Breast Imaging Reporting and Data System.

Among premenopausal women, no significant association between BMI and the risk of breast cancer was found in any of the BI-RADS categories after adjusting for other covariates (Figure 3; eTable 3 in the Supplement). However, among postmenopausal women, a high BMI was associated with a subsequent increased risk of breast cancer in total and across all breast density categories after adjusting for other potential confounders. Among postmenopausal women in BI-RADS category 1, those with obesity had a 2.45-fold (95% CI, 1.87-3.21) increased risk of breast cancer relative to women in the underweight category. Among postmenopausal women in high BI-RADS categories, a high level of obesity was associated with an increased risk of breast cancer compared with being underweight; however, the strength of the association was low among women with high breast density.

### Combined Associations of Breast Density and BMI With Breast Cancer Risk

Among premenopausal women, those in BI-RADS categories 1 and 2 did not show significant associations with breast cancer risk, irrespective of obesity status, compared with those in BI-RADS category 1 who were in the underweight category (Table 2). However, a significantly high breast cancer risk was found among women in BI-RADS categories 3 and 4 at all obesity levels within the same BI-RADS categories. For example, in BI-RADS category 3, the aRR in the underweight group was 1.47 (95% CI, 1.02-2.12), in the normal weight group was 1.57 (95% CI, 1.10-2.23), in the overweight group was 1.60 (95% CI, 1.12-2.27), and in the obese group was 1.63 (95% CI, 1.14-2.33). This finding suggests that breast density is an important risk factor for premenopausal women. Premenopausal women in BI-RADS category 4 with obesity had an approximately 2-fold higher risk of breast cancer than women in BI-RADS category 1 who were in the underweight category (aRR, 2.01; 95% CI, 1.40-2.88).

**Table 2.** Interactive Effects and Additive Interactions of BMI and Mammographic Breast Density on Breast Cancer Risk

Breast density	BMI category			
	Underweight	Normal	Overweight	Obese
**Premenopausal women**				
Univariate model (RR [95% CI])				
BI-RADS 1	1 [Reference]	0.91 (0.63-1.30)	0.83 (0.58-1.19)	0.73 (0.51-1.06)
BI-RADS 2	1.06 (0.70-1.61)	1.03 (0.72-1.46)	1.05 (0.73-1.49)	1.10 (0.77-1.57)
BI-RADS 3	1.40 (0.97-2.02)	1.52 (1.07-2.16)	1.58 (1.11-2.25)	1.62 (1.13-2.31)
BI-RADS 4	1.77 (1.23-2.53)	1.81 (1.27-2.58)	1.91 (1.34-2.72)	1.98 (1.38-2.84)
Additive interaction	0.57 (0.39-0.75)			
Multiplicative interaction	1.04 (1.02-1.06)			
Multivariate model (aRR [95% CI])				
BI-RADS 1	1 [Reference]	0.90 (0.63-1.29)	0.79 (0.55-1.13)	0.71 (0.49-1.02)
BI-RADS 2	1.09 (0.72-1.66)	1.04 (0.73-1.48)	1.04 (0.73-1.48)	1.09 (0.76-1.57)
BI-RADS 3	1.47 (1.02-2.12)	1.57 (1.10-2.23)	1.60 (1.12-2.27)	1.63 (1.14-2.33)
BI-RADS 4	1.85 (1.29-2.65)	1.88 (1.32-2.68)	1.95 (1.37-2.77)	2.01 (1.40-2.88)
Additive interaction	0.53 (0.35-0.71)			
Multiplicative interaction	1.04 (1.02-1.06)			
**Postmenopausal women**				
Univariate model (RR [95% CI])				
BI-RADS 1	1 [Reference]	1.61 (1.23-2.11)	2.07 (1.59-2.70)	2.75 (2.10-3.59)
BI-RADS 2	2.11 (1.51-2.95)	3.10 (2.37-4.04)	3.87 (2.97-5.05)	4.83 (3.69-6.31)
BI-RADS 3	3.67 (2.71-4.97)	4.71 (3.61-6.15)	5.58 (4.28-7.28)	6.90 (5.27-9.04)
BI-RADS 4	4.50 (3.29-6.16)	5.81 (4.44-7.60)	6.31 (4.82-8.26)	7.15 (5.35-9.55)
Additive interaction	1.84 (1.37-2.32)			
Multiplicative interaction	0.99 (0.97-1.01)			
Multivariate model (aRR [95% CI])				
BI-RADS 1	1 [Reference]	1.57 (1.20-2.05)	2.00 (1.53-2.61)	2.64 (2.02-3.45)
BI-RADS 2	2.00 (1.44-2.80)	2.88 (2.21-3.76)	3.61 (2.77-4.72)	4.49 (3.44-5.88)
BI-RADS 3	3.28 (2.42-4.45)	4.23 (3.24-5.52)	5.05 (3.87-6.59)	6.23 (4.76-8.17)
BI-RADS 4	3.88 (2.83-5.31)	5.06 (3.87-6.63)	5.58 (4.26-7.32)	6.31 (4.72-8.43)
Additive interaction	1.68 (1.26-2.10)			
Multiplicative interaction	0.99 (0.97-1.01)			

Abbreviations: aRR, adjusted relative risk; BI-RADS, Breast Imaging Reporting and Data System; BMI, body mass index (calculated as weight in kilograms divided by height in meters squared); RR, relative risk.

Compared with postmenopausal women in BI-RADS category 1 who were in the underweight category, women in BI-RADS category 1 with obesity (aRR, 2.64; 95% CI, 2.02-3.45) and women in BI-RADS category 4 who were in the underweight category (aRR, 3.88; 95% CI, 2.83-5.31) showed an increased risk of breast cancer (Table 2); this finding suggests that both higher breast density and higher BMI were associated with a higher breast cancer risk. The combination of BI-RADS category 4 and obesity was associated with the highest risk of breast cancer, which was approximately 6-fold higher than that of the reference BMI group (aRR, 6.31; 95% CI, 4.72-8.34).

A high breast density and high BMI had a significant positive interaction on the additive scale in both premenopausal and postmenopausal women, especially the latter (Table 2). The adjusted RERI was 0.53 (95% CI, 0.35-0.71) in premenopausal women and 1.68 (95% CI, 1.26-2.10) in postmenopausal women. A marginally positive multiplicative interaction was found among premenopausal women (adjusted multiplicative interaction, 1.04; 95% CI, 1.02-1.06); however, no significant multiplicative interaction was found among postmenopausal women (adjusted multiplicative interaction, 0.99; 95% CI, 0.97-1.01).

## Discussion

This population-based cohort study found that breast density was independently associated with breast cancer risk for premenopausal and postmenopausal women; however, obesity was associated with breast cancer risk only for postmenopausal women. Considering the combined associations of breast density and obesity, women with obesity in BI-RADS category 4 showed the highest risk of breast cancer among both premenopausal and postmenopausal women, which was 2-fold higher for premenopausal women and 6-fold higher for postmenopausal women than that for women in BI-RADS category 1 who were in the underweight category. A positive synergistic association in terms of additive interaction was suggested in both groups, especially for postmenopausal women. This study confirmed the association between dense breast tissue and increased breast cancer risk previously found in other populations^2,8,18^ and for Korean women.^3,19^ Although increased adiposity was not associated with premenopausal breast cancer risk, it showed a strong association with an increased risk of postmenopausal breast cancer, which was also found in our study.^20,21^

Previous studies on the interactive associations between breast density and BMI with breast cancer risk provide mixed evidence. A case-control study found that women with a breast density of 76% to 100% had a 5-fold higher risk of breast cancer than those with a breast density of 0% to 10%; moreover, breast density had a significant multiplicative interaction with BMI.^22^ Other case-control studies in the US^12^ and Japan^23^ also reported that breast density was associated with an increased risk of premenopausal and postmenopausal breast cancers, and the association was strong in women with overweight or obesity, showing a multiplicative interaction. In contrast, no multiplicative interaction association between breast density and BMI with breast cancer risk was reported in a pooled analysis of 4 case-control studies^6^ and a study on postmenopausal women from the Nurses’ Health Study.^8^ A recent nested case-control study reported that the combination of overweight or obesity and elevated breast density was associated with a higher risk of premenopausal breast cancer.^9^ In particular, a high association was found with estrogen receptor–negative subtypes (odds ratio, 2.17; 95% CI, 1.50-3.16); however, no interactions between breast density and BMI were detected in postmenopausal women. Although the interactive associations of breast density and BMI with breast cancer risk have been studied previously, most studies reported only multiplicative interactions despite the necessity of assessing interactions in both multiplicative and additive scales.^24,25^ In our study, we assessed both scale interactions and identified that, despite the small or nonsignificant multiplicative interaction of BMI and breast density with breast cancer risk, positive interactions were observed in both premenopausal and postmenopausal women. Considering that the additive interaction model is a relevant public health measure,^24,25^ both breast density and adiposity might need to be considered in the identification of high-risk individuals in a population-based screening setting.

Percentage of breast tissue density and adiposity are individually associated with an increased risk of breast cancer but are negatively associated with one another.^26,27^ Thus, the biological mechanisms underlying the interaction between breast density and BMI with breast cancer risk are complex and poorly understood. Breast cancer growth involves neoplastic transformation of breast epithelial cells into malignant cells. Given that epithelial cells are surrounded by adipose tissue, inflammation in breast adipose tissue is thought to play an important role in breast tumor development. The inflammatory effects of adiposity are mediated through increases in breast density, which may explain the combined associations of BMI and breast density.^26,28^ Another mechanism is associated with the aromatase activity of body fat, which is a significant source of endogenous estrogens that promote breast tissue proliferation.^26^ These complex biological associations of adiposity and breast density may explain the different interaction measures in terms of additive or multiplicative scales. Studies have also reported that the absence of interaction on either scale would be common^24,25^; however, there would always be an interaction on at least 1 scale when both exposures were associated with breast cancer risk with sufficient statistical power.

### Strengths and Limitations

This study has some strengths, including its large national cohort design and prospective ascertainment of breast cancer development. Most previous studies have investigated the interaction between BMI and breast density using a multiplicative approach. However, considering that additive interactions would be relevant for assessing the public health effect of an interaction,^17^ the positive additive interaction for both premenopausal and postmenopausal women found in our study might be informative. To our knowledge, this study is the first to comprehensively investigate the combined association of breast density and BMI with breast cancer risk among women in Asian countries based on population-based breast screening settings, considering both additive and multiplicative interactions. Thus, this study provides evidence from real-world data on breast cancer risk, and the findings are generalizable to East Asian women, who tend to have a higher breast density than Western women.^29^ In addition, given the consistent findings from this study with previous studies conducted in Western countries,^9,12^ our results could also be applied to Western populations of women with increasing obesity.

This study also has some limitations. It included a population of women aged 40 years or older, which prevented us from examining breast cancer risk in younger groups. Furthermore, breast density was subjectively interpreted. Although the BI-RADS classification has been widely used for breast density classification, the results might vary depending on the ability and experience of radiologists. However, in Korea, a mammography education program to standardize the performance of radiologists is available and thus might increase the reproducibility of the density measures.^30^ Interradiologist variability was assessed in randomly selected digital mammograms from the Korean National Breast Cancer Screening Program, which reported an interradiologist variability of 0.83, indicating very high agreement.^31^ Although information on the reliability of the BI-RADS density categories between radiologists in Korea remained limited, several previous studies showed high agreement in assessing the film BI-RADs density categories between radiologists.^32,33^ Another limitation of this study was the relatively small numbers of breast cancer cases in underweight women in BI-RADS density category 1 in both premenopausal women (31 cases among 4353 participants) and postmenopausal women (55 cases among 28 167 participants).

## Conclusions

This study found that elevated breast density and having overweight or obesity are independently associated with an increased risk of breast cancer and interact synergistically to augment breast cancer risk for both premenopausal and postmenopausal women in terms of additive interactions. In addition, strong multiplicative interaction associations of BMI and breast density with breast cancer risk were found for postmenopausal women. Recent work suggests that notification of breast density should be combined with breast cancer risk to identify women at the highest risk so that they can undergo supplemental imaging screening and engage in discussions with their health care professionals.^34,35^ Our findings suggest that breast density notification should be provided not as a stand-alone risk factor but as an adjunct factor with BMI for risk stratification in population-based mammographic screening settings for public health significance.

## Notes

Supplement. **eTable 1.** Frequency of Breast Cancer and Non-Breast Cancer Development by BMI Levels and Breast Density Categories **eTable 2.** Univariate Poisson Regression Model for Association of Mammographic Breast Density With Breast Cancer Risk by BMI Levels **eTable 3.** Univariate Poisson Regression Model for Association of BMI Levels With Breast Cancer Risk by Mammographic Breast Density Category

## References

[1] Sung H, Ferlay J, Siegel RL, . Global cancer statistics 2020: GLOBOCAN estimates of incidence and mortality worldwide for 36 cancers in 185 countries. CA Cancer J Clin. 2021;71(3):209-249. doi:10.3322/caac.21660 33538338

[2] Pettersson A, Graff RE, Ursin G, . Mammographic density phenotypes and risk of breast cancer: a meta-analysis. J Natl Cancer Inst. 2014;106(5):dju078. doi:10.1093/jnci/dju078 24816206 PMC4568991

[3] Kim S, Park B. Association between changes in mammographic density category and the risk of breast cancer: a nationwide cohort study in East-Asian women. Int J Cancer. 2021;148(11):2674-2684. doi:10.1002/ijc.33455 33368233

[4] Schoemaker MJ, Nichols HB, Wright LB, ; Premenopausal Breast Cancer Collaborative Group. Association of body mass index and age with subsequent breast cancer risk in premenopausal women. JAMA Oncol. 2018;4(11):e181771. doi:10.1001/jamaoncol.2018.1771 29931120 PMC6248078

[5] Neuhouser ML, Aragaki AK, Prentice RL, . Overweight, obesity, and postmenopausal invasive breast cancer risk: a secondary analysis of the Women’s Health Initiative randomized clinical trials. JAMA Oncol. 2015;1(5):611-621. doi:10.1001/jamaoncol.2015.1546 26182172 PMC5070941

[6] Conroy SM, Woolcott CG, Koga KR, . Mammographic density and risk of breast cancer by adiposity: an analysis of four case-control studies. Int J Cancer. 2012;130(8):1915-1924. doi:10.1002/ijc.26205 21630258 PMC3254813

[7] Wong CS, Lim GH, Gao F, . Mammographic density and its interaction with other breast cancer risk factors in an Asian population. Br J Cancer. 2011;104(5):871-874. doi:10.1038/sj.bjc.6606085 21245860 PMC3048202

[8] Yaghjyan L, Colditz GA, Rosner B, Tamimi RM. Mammographic breast density and breast cancer risk: interactions of percent density, absolute dense, and non-dense areas with breast cancer risk factors. Breast Cancer Res Treat. 2015;150(1):181-189. doi:10.1007/s10549-015-3286-6 25677739 PMC4372799

[9] Shieh Y, Scott CG, Jensen MR, . Body mass index, mammographic density, and breast cancer risk by estrogen receptor subtype. Breast Cancer Res. 2019;21(1):48. doi:10.1186/s13058-019-1129-9 30944014 PMC6448282

[10] Boyd NF, Martin LJ, Sun L, . Body size, mammographic density, and breast cancer risk. Cancer Epidemiol Biomarkers Prev. 2006;15(11):2086-2092. doi:10.1158/1055-9965.EPI-06-0345 17119032

[11] Duffy SW, Morrish OWE, Allgood PC, . Mammographic density and breast cancer risk in breast screening assessment cases and women with a family history of breast cancer. Eur J Cancer. 2018;88:48-56. doi:10.1016/j.ejca.2017.10.022 29190506 PMC5768323

[12] Engmann NJ, Scott CG, Jensen MR, . Combined effect of volumetric breast density and body mass index on breast cancer risk. Breast Cancer Res Treat. 2019;177(1):165-173. doi:10.1007/s10549-019-05283-z 31129803 PMC6640105

[13] Conroy SM, Woolcott CG, Koga KR, . Mammographic density and risk of breast cancer by adiposity: an analysis of four case-control studies. Int J Cancer. 2012;130(8):1915-1924. doi:10.1002/ijc.26205 21630258 PMC3254813

[14] Lee J, Lee JS, Park SH, Shin SA, Kim K. Cohort profile: the National Health Insurance Service–National Sample Cohort (NHIS-NSC), South Korea. Int J Epidemiol. 2017;46(2):e15. doi:10.1093/ije/dyv319 26822938

[15] Kim S, Kwon S. Impact of the policy of expanding benefit coverage for cancer patients on catastrophic health expenditure across different income groups in South Korea. Soc Sci Med. 2015;138:241-247. doi:10.1016/j.socscimed.2015.06.012 26123883

[16] World Health Organization. The Asia-Pacific Perspective: Redefining Obesity and Its Treatment. World Health Organization; 2000.

[17] VanderWeele T. Explanation in Causal Inference: Methods for Mediation and Interaction. Oxford University Press; 2015.

[18] Boyd NF, Guo H, Martin LJ, . Mammographic density and the risk and detection of breast cancer. N Engl J Med. 2007;356(3):227-236. doi:10.1056/NEJMoa062790 17229950

[19] Park B, Cho HM, Lee EH, . Does breast density measured through population-based screening independently increase breast cancer risk in Asian females? Clin Epidemiol. 2017;10:61-70. doi:10.2147/CLEP.S144918 29343988 PMC5749627

[20] Smith SG, Sestak I, Morris MA, . The impact of body mass index on breast cancer incidence among women at increased risk: an observational study from the International Breast Intervention Studies. Breast Cancer Res Treat. 2021;188(1):215-223. doi:10.1007/s10549-021-06141-7 33656637 PMC8233270

[21] Cheraghi Z, Poorolajal J, Hashem T, Esmailnasab N, Doosti Irani A. Effect of body mass index on breast cancer during premenopausal and postmenopausal periods: a meta-analysis. PLoS One. 2012;7(12):e51446. doi:10.1371/journal.pone.0051446 23236502 PMC3517558

[22] Wong CS, Lim GH, Gao F, . Mammographic density and its interaction with other breast cancer risk factors in an Asian population. Br J Cancer. 2011;104(5):871-874. doi:10.1038/sj.bjc.6606085 21245860 PMC3048202

[23] Nishiyama K, Taira N, Mizoo T, . Influence of breast density on breast cancer risk: a case control study in Japanese women. Breast Cancer. 2020;27(2):277-283. doi:10.1007/s12282-019-01018-6 31650498

[24] VanderWeele TJ, Knol MJ. A tutorial on interaction. Epidemiol Methods. 2014;3(1):33-72. doi:10.1515/em-2013-0005

[25] Rothman KJ, Greenland S, Lash TL. Modern Epidemiology. Vol 3. Wolters Kluwer Health/Lippincott Williams & Wilkins Philadelphia; 2008.

[26] Soguel L, Durocher F, Tchernof A, Diorio C. Adiposity, breast density, and breast cancer risk: epidemiological and biological considerations. Eur J Cancer Prev. 2017;26(6):511-520. doi:10.1097/CEJ.0000000000000310 27571214 PMC5627530

[27] Hjerkind KV, Ellingjord-Dale M, Johansson ALV, . Volumetric mammographic density, age-related decline, and breast cancer risk factors in a national breast cancer screening program. Cancer Epidemiol Biomarkers Prev. 2018;27(9):1065-1074. doi:10.1158/1055-9965.EPI-18-0151 29925631

[28] Bhardwaj P, Brown KA. Obese adipose tissue as a driver of breast cancer growth and development: update and emerging evidence. Front Oncol. 2021;11:638918. doi:10.3389/fonc.2021.638918 33859943 PMC8042134

[29] Jo HM, Lee EH, Ko K, ; Alliance for Breast Cancer Screening in Korea (ABCS-K). Prevalence of women with dense breasts in Korea: results from a nationwide cross-sectional study. Cancer Res Treat. 2019;51(4):1295-1301. doi:10.4143/crt.2018.297 30699499 PMC6790853

[30] Lee EH, Jun JK, Jung SE, Kim YM, Choi N. The efficacy of mammography boot camp to improve the performance of radiologists. Korean J Radiol. 2014;15(5):578-585. doi:10.3348/kjr.2014.15.5.578 25246818 PMC4170158

[31] Jo HM, Song S, Lee EH, . Interpretive volume and inter-radiologist agreement on assessing breast density. J Korean Soc Breast Screening. 2018;15:15-22.

[32] Winkel RR, von Euler-Chelpin M, Nielsen M, . Inter-observer agreement according to three methods of evaluating mammographic density and parenchymal pattern in a case control study: impact on relative risk of breast cancer. BMC Cancer. 2015;15:274. doi:10.1186/s12885-015-1256-3 25884160 PMC4397728

[33] Spayne MC, Gard CC, Skelly J, Miglioretti DL, Vacek PM, Geller BM. Reproducibility of BI-RADS breast density measures among community radiologists: a prospective cohort study. Breast J. 2012;18(4):326-333. doi:10.1111/j.1524-4741.2012.01250.x 22607064 PMC3660069

[34] Kerlikowske K, Sprague BL, Tosteson ANA, . Strategies to identify women at high risk of advanced breast cancer during routine screening for discussion of supplemental imaging. JAMA Intern Med. 2019;179(9):1230-1239. doi:10.1001/jamainternmed.2019.1758 31260054 PMC6604099

[35] Bakker MF, de Lange SV, Pijnappel RM, ; DENSE Trial Study Group. Supplemental MRI screening for women with extremely dense breast tissue. N Engl J Med. 2019;381(22):2091-2102. doi:10.1056/NEJMoa1903986 31774954

