# Supplementary material for: Association of the Interaction Between Mammographic Breast Density, Body Mass Index, and Menopausal Status With Breast Cancer Risk Among Korean Women
Source: JAMA Netw Open. 2021 Dec 23;4(12):e2139161. doi: 10.1001/jamanetworkopen.2021.39161 (PMC8703253; doi:10.1001/jamanetworkopen.2021.39161)
Supplement: Supplement. — eTable 1. Frequency of Breast Cancer and Non-Breast Cancer Development by BMI Levels and Breast Density Categories eTable 2. Univariate Poisson Regression Model for Association of Mammographic Breast Density With Breast Cancer Risk by BMI Levels eTable 3. Univariate Poisson Regression Model for Association of BMI Levels With Breast Cancer Risk by Mammographic Breast Density Category [file jamanetwopen-e2139161-s001.pdf]

Supplementary Online Content

Tran TXM, Moon SG, Kim S, Park B. Association of the interaction between mammographic breast density, body mass index, and menopausal status with breast cancer risk among Korean women. *JAMA Netw Open.* 2021;4(12):e2139161. doi:10.1001/jamanetworkopen.2021.39161

eTable 1. Frequency of Breast Cancer and Non-Breast Cancer Development by BMI Levels and Breast Density Categories

eTable 2. Univariate Poisson Regression Model for Association of Mammographic Breast Density with Breast Cancer Risk by BMI Levels

eTable 3. Univariate Poisson Regression Model for Association of BMI Levels with Breast Cancer Risk by Mammographic Breast Density Category

This supplementary material has been provided by the authors to give readers additional information about their work.

© 2021 Tran TXM et al. *JAMA Network Open.*

eTable 1. Frequency of Breast Cancer and Non-Breast Cancer Development by BMI Levels and Breast Density Categories

BMI level	BI-RADS 1		BI-RADS 2		BI-RADS 3		BI-RADS 4	
	**Breast cancer**	**Non-breast cancer**	**Breast cancer**	**Non-breast cancer**	**Breast cancer**	**Non-breast cancer**	**Breast cancer**	**Non-breast cancer**
	**n (%)**	**n (%)**	**n (%)**	**n (%)**	**n (%)**	**n (%)**	**n (%)**	**n (%)**
**Premenopausal women**								
Underweight	31 (2)	4 322 (2)	76 (2)	9 978 (2)	390 (3)	38 651 (3)	772 (6)	60 582 (6)
Normal	595 (39)	91 425 (36)	1 746 (39)	237 258 (39)	7606 (49)	695 052 (50)	7 672 (59)	586 721 (60)
Overweight	657 (43)	110 611 (43)	1 981 (44)	263 600 (44)	6004 (39)	526 486 (38)	3 899 (30)	282 488 (29)
Obese	262 (17)	49 884 (19)	724 (16)	91 889 (15)	1398 (9)	119 921 (9)	653 (5)	45 607 (5)
Total	1 545 (100)	256 242 (100)	4 527 (100)	602 725 (100)	15398 (100)	1 380 110 (100)	12 996 (100)	975 398 (100)
**Postmenopausal women**								
Underweight	55 (1)	28 112 (2)	94 (1)	22 688 (2)	172 (2)	23 852 (2)	134 (4)	15 116 (5)
Normal	1 465 (22)	463 602 (28)	2 830 (28)	465 312 (34)	3952 (38)	425 797 (43)	1 810 (50)	157 754 (51)
Overweight	3 443 (52)	848 523 (51)	5 333 (53)	700 422 (51)	4962 (48)	450 537 (45)	1 427 (39)	114 324 (37)
Obese	1 704 (26)	316 085 (19)	1 877 (19)	197 231 (14)	1280 (12)	93 667 (9)	278 (8)	19 635 (6)
Total	6 667 (100)	16 56 322 (100)	10 134 (100)	1 385 653 (100)	10366 (100)	993 853 (100)	3 649 (100)	306 829 (100)

© 2021 Tran TXM et al. *JAMA Network Open.*

eTable 2. Univariate Poisson Regression Model for Association of Mammographic Breast Density with Breast Cancer Risk by BMI Levels

BMI levels	BI-RADS category	Premenopausal women		Postmenopausal women	
		RR	95%CI	RR	95%CI
**Total**					
	BI-RADS 1	1 [Reference]		1 [Reference]	
	BI-RADS 2	1.24	1.17 - 1.32	1.81	1.76 - 1.87
	BI-RADS 3	1.84	1.75 - 1.94	2.57	2.50 - 2.66
	BI-RADS 4	2.19	2.08 - 2.31	2.93	2.82 - 3.05
**Underweight**					
	BI-RADS 1	1 [Reference]		1 [Reference]	
	BI-RADS 2	1.06	0.70 - 1.61	2.11	1.52 - 2.95
	BI-RADS 3	1.40	0.97 - 2.02	3.67	2.71 - 4.96
	BI-RADS 4	1.77	1.24 - 2.53	4.50	3.29 - 6.16
**Normal**					
	BI-RADS 1	1 [Reference]		1 [Reference]	
	BI-RADS 2	1.13	1.03 - 1.24	1.92	1.80 - 2.04
	BI-RADS 3	1.67	1.54 - 1.82	2.92	2.75 - 3.10
	BI-RADS 4	2.00	1.84 - 2.17	3.60	3.36 - 3.86
**Overweight**					
	BI-RADS 1	1 [Reference]		1 [Reference]	
	BI-RADS 2	1.26	1.16 - 1.38	1.87	1.79 - 1.95
	BI-RADS 3	1.91	1.76 - 2.07	2.70	2.58 - 2.81
	BI-RADS 4	2.31	2.12 - 2.50	3.05	2.87 - 3.24
**Obese**					
	BI-RADS 1	1 [Reference]		1 [Reference]	
	BI-RADS 2	1.50	1.30 - 1.72	1.76	1.65 - 1.88
	BI-RADS 3	2.21	1.93 - 2.52	2.51	2.34 - 2.70
	BI-RADS 4	2.70	2.34 - 3.12	2.60	2.30 - 2.95

Abbreviations: BMI: body mass index (calculated as weight in kilograms divided by height in meters squared); RR: relative risk, RR: relative risk; CI: confidence interval

© 2021 Tran TXM et al. *JAMA Network Open.*

eTable 3. Univariate Poisson Regression Model for Association of BMI Levels with Breast Cancer Risk by Mammographic Breast Density Category

**BI-RADS categories**	**BMI levels**	**Premenopausal women**		**Postmenopausal women**	
		**RR**	**95% CI**	**RR**	**95% CI**
**Total**					
	Underweight	1 [Reference]		1 [Reference]	
	Normal	0.98	0.92 - 1.04	1.31	1.19 - 1.44
	Overweight	0.95	0.90 - 1.01	1.41	1.29 - 1.55
	Obese	0.89	0.83 - 0.95	1.61	1.47 - 1.78
**BI-RADS 1**					
	Underweight	1 [Reference]		1 [Reference]	
	Normal	0.91	0.63 - 1.30	1.61	1.23 - 2.11
	Overweight	0.83	0.58 - 1.19	2.07	1.59 - 2.70
	Obese	0.73	0.51 - 1.06	2.75	2.10 - 3.59
**BI-RADS 2**					
	Underweight	1 [Reference]		1 [Reference]	
	Normal	0.97	0.77 - 1.21	1.47	1.19 - 1.80
	Overweight	0.99	0.79 - 1.24	1.83	1.49 - 2.24
	Obese	1.03	0.82 - 1.31	2.28	1.86 - 2.81
**BI-RADS 3**					
	Underweight	1 [Reference]		1 [Reference]	
	Normal	1.08	0.98 - 1.20	1.28	1.10 - 1.50
	Overweight	1.13	1.02 - 1.25	1.52	1.31 - 1.77
	Obese	1.15	1.03 - 1.29	1.88	1.61 - 2.21
**BI-RADS 4**					
	Underweight	1 [Reference]		1 [Reference]	
	Normal	1.03	0.95 - 1.10	1.29	1.08 - 1.54
	Overweight	1.08	1.00 - 1.17	1.40	1.18 - 1.67
	Obese	1.12	1.01 - 1.24	1.59	1.29 - 1.95

Abbreviations: BMI: body mass index (calculated as weight in kilograms divided by height in meters squared); RR: relative risk, RR: relative risk; CI: confidence interval

© 2021 Tran TXM et al. *JAMA Network Open.*
